# Aerobic Exercise Attenuates Acute Lung Injury Through NET Inhibition

**DOI:** 10.3389/fimmu.2020.00409

**Published:** 2020-03-19

**Authors:** Yue Shi, Tingting Liu, David C. Nieman, Yanqiu Cui, Fei Li, Luyu Yang, Hui Shi, Peijie Chen

**Affiliations:** ^1^School of Kinesiology, Shanghai University of Sport, Shanghai, China; ^2^Department of Rheumatology and Immunology, Ruijin Hospital, Shanghai Jiao Tong University School of Medicine, Shanghai, China; ^3^North Carolina Research Campus, Appalachian State University, Kannapolis, NC, United States; ^4^School of Physical Education and Sport Training, Shanghai University of Sport, Shanghai, China; ^5^Department of General Surgery, Cancer Metastasis Institute, Huashan Hospital, Fudan University, Shanghai, China

**Keywords:** acute lung injury, aerobic exercise, neutrophil extracellular traps, alveolar macrophages, inflammation

## Abstract

**Introduction:** Aerobic exercise improves lung inflammation in acute lung injury (ALI), but its mechanism remains unknown. Neutrophil extracellular traps (NETs) play an important role in LPS-induced ALI, and a positive correlation exists between NET formation and proinflammatory macrophage polarization. This study investigated whether aerobic exercise reduces the pro-inflammatory polarization of alveolar macrophages (AMs) by inhibiting the excessive release of NETs and then alleviating the inflammatory response of ALI.

**Methods:** C57BL/6 male mice were randomly divided into four groups: sedentary group (CON), sedentary and extra-pulmonary LPS injection group (LPS), 5-weeks aerobic training intervention and LPS injection group (EXE+LPS), and DNase I plus LPS injection group (DNase+LPS). Twenty-four hours after drug injection, bronchoalveolar lavage fluid (BALF), AM, and lung tissues were obtained to detect inflammatory responses, NET formation, macrophage polarization, and protein activation. In the *in vitro* study, a murine AM cell line, designated MH-S, was stimulated with LPS, purified NETs, and NETs plus DNase I.

**Results:** EXE+LPS and DNase+LPS mice exhibited reduced neutrophil infiltration, decreased NET release, and lower pro-inflammatory polarization of AM compared with LPS mice. Subsequently, Western blot showed inhibition of the phosphorylation of MAPK and NF-κB proteins of AMs in EXE+LPS and DNase+LPS mice compared with LPS mice. Lastly, stimulation of MH-S cells by NETs revealed a trend for pro-inflammatory cell polarization, with NF-κB protein activation at 8 h and ERK1/2 activation at 1, 2, and 8 h.

**Conclusions:** Aerobic exercise alleviated ALI through NET-induced AM pro-inflammatory polarization involving ERK1/2 and NF-κB signaling.

## Introduction

Acute lung injury (ALI) is a common clinical respiratory illness characterized by acute and progressive respiratory insufficiency caused by a variety of causes. The main clinical features are respiratory distress and progressive hypoxemia (1). Due to the complex etiology and pathogenesis, the current morbidity mortality rate remains high. Excessive airway inflammatory response is regarded as the underlying cause of ALI and, when uncontrolled, leads to acute respiratory distress syndrome (ARDS) and even respiratory failure (2). Lipopolysaccharide (LPS), a main component of the gram-negative bacteria cell wall, has been identified as a key factor that activates inflammatory signaling cascades in ALI/ARDS development (3). Intratracheal instillation and intraperitoneal injection of LPS in rodents are effective methods for establishing ALI models. Aerobic exercise training for 5 weeks may inhibit extra-pulmonary LPS-induced lung inflammation by attenuating inflammatory cytokines and oxidative stress markers via IL-10 production (4). Aerobic exercise training has been widely accepted as having a positive influence on immune function and surveillance. However, the underlying molecular and signaling mechanisms of how aerobic exercise training may counter the ALI inflammation response is not well-understood.

Neutrophil extracellular traps (NETs) are released by activated neutrophils and are composed mainly of DNA, histones, myeloperoxidase (5), and neutrophil elastase (NE). The extracellular web-like structures can effectively trap invading pathogens and utilize highly localized concentrations of antimicrobial peptides to degrade virulence elements (6). Although NET formation is a potent process for host defense against foreign pathogen invasion, excessive NET release can also have adverse consequences. Proteins and dsDNA components located in NETs may be the source of key autoantigens, which lead to an inflammatory cascade in local tissues and the blood compartment. These autoantigens are involved in a variety of autoimmunity diseases such as rheumatoid arthritis (RA), systemic lupus erythematosus (SLE), psoriasis, and gout (7).

Recently, NET formation was found in LPS-induced lung inflammation and was degraded by aerosolized DNase I treatment in mice (8). The inhibition of NETs was correlated with suppression of the TLR4/NF-κB signaling pathway (9). In addition, alveolar M1 and M2 macrophages exhibit pro-inflammatory and anti-inflammatory properties, respectively. In response to LPS treatment in ALI mice, alveolar macrophages (AMs) undergo M1 activation, characterized by the expression and secretion of pro-inflammatory factors, such as TNF-α, IL-1β, and inducible nitric oxide synthase (iNOS) (10). NET levels in ARDS patients have been positively correlated with M1-like macrophage polarization (11). These studies support a linkage between NET formation and pro-inflammatory macrophage polarization, but direct evidence is lacking. Additionally, little is known regarding the beneficial role aerobic exercise training may play in attenuating AM pro-inflammatory polarization and NET formation, especially within the context of LPS-induced ALI.

In this study with ALI mice, we demonstrated that 5 weeks of aerobic treadmill exercise reduced LPS-induced lung inflammation and inhibited NET formation, similar to DNase I treatment. This beneficial response was shown to linked to alterations in the mitogen-activated protein kinase (MAPK) (ERK1/2, JNK and p38) and NF-κB signaling pathways. Additional cell culture studies with the murine AM cell line MH-S showed that NET formation was coupled to pro-inflammatory macrophage polarization and inflammation.

## Methods

### Animals

Seven-week-old male C57BL/6 mice, weighing 20–25 g, were purchased from the GemPharmatec Company (Nanjing, China) and fed for 1 week to allow for acclimatization. Mice were housed in a constant temperature at 25°C with a 12-h dark/light cycle and free access to standard pellets and water. The mice were randomly divided into four groups: control (CON), intraperitoneal injection of LPS alone (LPS), 5 weeks of aerobic treadmill running plus intraperitoneal injection of LPS (EXE+LPS), and intraperitoneal injection of LPS and DNase I (DNase+LPS). All experimental protocols of this study were approved by the Ethics Committee of Shanghai University of Sport. Study procedures with the mice were in compliance with standards established by the Ethics Committee for Animal Experimentation of Shanghai University of Sport and the Guide for the Care and Use of Laboratory Animals (Institute for Laboratory Animal Research, USA). The data shown in the experiment were based on three repeated experiments.

### Aerobic Exercise Protocol

The aerobic treadmill running training protocol was based on a maximal running capacity test conducted before the start of formal research, as reported previously (12). Maximum running capacity was determined as follows: 10 min warm-up (5% grade, 10 m/min) followed by an increase in the treadmill speed (3 m/min each 3 min) until exhaustion and an inability to run even after repeated mechanical stimuli. Maximal exercise capacity (100%) was set as the maximum speed reached by each mouse. EXE+LPS group mice were then trained using moderate intensity exercise (60% of average maximum speed) for 60 min/day, 5 days/week for 5 weeks (4); mice in CON, LPS, and DNase+LPS groups were all housed in the standard cages for the 5 weeks with no intervention.

### Animal Exposure to LPS and DNase I Administration

**Figure 1:**
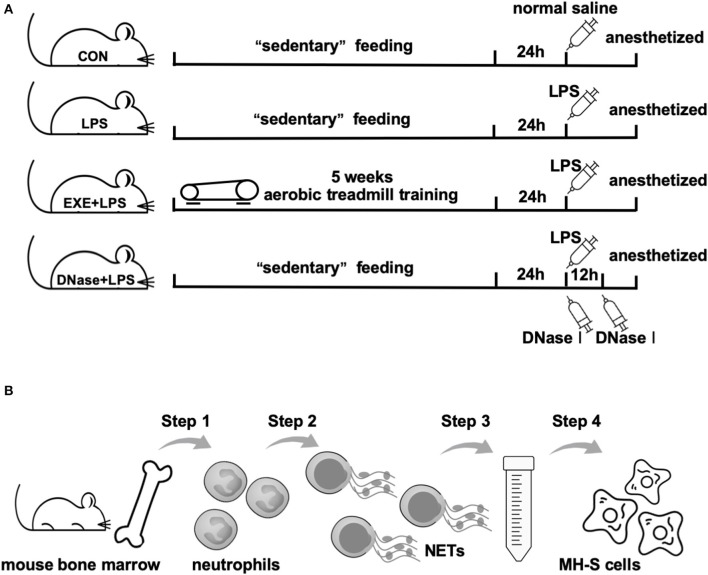
Operational schematics of *in vivo* and *in vitro* experiments. **(A)** Schematic diagram of the time axis of intervention in each group of mice. Mice in CON, LPS, and DNase+LPS groups were housed for 5 weeks of “sedentary” feeding and mice in EXE+LPS group were engaged in 5 weeks of aerobic treadmill training. Twenty-four hours after last training, all mice except the CON group were anesthetized and received intraperitoneal administration of 100 μl of *Escherichia coli* LPS diluted in saline at a dose of 1 μg/μl. Mice in the CON group were submitted to intraperitoneal injection of saline alone, while DNase+LPS mice received aerosolized DNase I 10 μg diluted in 100 μl normal saline at 0 and 12 h after intraperitoneal LPS injection. Then, 24 h later, all mice were anesthetized with 2.5% chloral hydrate (0.1 ml/10 g), and the following analyses were performed. **(B)** General operation procedures of *in vitro* experiment: Step 1: Neutrophils were isolated from mouse bone marrow; Step 2: NET formation through PMA stimulation; Step 3: Extraction and purification of NETs by low- and high-speed centrifugation; Step 4: MH-S cells were stimulated with purified NETs to observe polarization reaction.

Twenty-four hours after the last treadmill run of EXE+LPS group mice, mice of LPS, EXE+LPS, and DNase+ LPS groups were anesthetized and received intraperitoneal administration of 100 μl of *Escherichia coli* LPS (055: B5; Sigma-Aldrich, St. Louis, MO) diluted in saline at a dose of 1 μg/μl. Mice in the CON group were submitted to intraperitoneal injection of saline alone. To investigate the role of DNase I on reducing lung inflammation response, DNase+LPS mice received aerosolized DNase I (Sigma-Aldrich, D5025) 10 μg diluted in 100 μl of normal saline at 0 and 12 h after intraperitoneal LPS injection. Then, 24 h later, all mice were anesthetized with 2.5% chloral hydrate (0.1 ml/10 g) and the following analyses were performed (Figure 1A).

### Collection of Bronchoalveolar Lavage Fluid (BALF)

Mice were anesthetized and the tracheae were cannulated by a 20-gauge catheter. The lungs were lavaged with 0.6 ml of ice-cold D-PBS for five consecutive washes. A total of 2.3 to 2.7 ml of BALF was recovered and centrifuged at 1,500 rpm, at 4°C, for 5 min. The supernatant was stored at −20°C for cytokine and MPO–DNA complex analysis. The cell pellet was resuspended in PBS for flow cytometry detection and acquisition of AMs.

### Cell Culture and Treatment

Primary AMs were collected from C57BL/6 mice by the differential adhesion method. MH-S cells (derived from mouse AMs) were obtained from American Type Culture Collection (ATCC) (Manassas, VA, USA). Cells were cultured in RPMI 1640 medium, supplemented with 10% fetal bovine serum and 1% penicillin-streptomycin (Gibco BRL, MD, USA) at 37°C in a 5% CO_2_ atmosphere. Cells were treated with media containing 100 ng/ml LPS, 1 ng/μl NETs, NETs plus DNase, and DNase alone for different time points. NETs plus DNase treatment was pre-processed as previously reported (13). NETs were digested for 15 min in 1 ml of RPMI with 10 U/ml DNase I at 37°C. Then, DNase was stopped with 2 mM EDTA. After incubation, the supernatant was collected for cytokine detection and the cells were collected for RT-PCR and Western blot analysis (Figure 1B).

### Histology, Mucus Staining, and Immunofluorescence

The left lung lobes were fixed in 4% PFA for 48 h and then routinely processed to paraffin blocks and sectioned at ~4 μm. H&E staining of lung tissue sections was performed to evaluate the inflammatory response. The tissue sections were observed under a light microscope for the lung histopathology. To detect neutrophils and macrophages in lung tissue, paraffin-embedded mouse lung sections were permeabilized with 0.2% Triton X-100 in phosphate-buffered saline (PBS) for 15 min and blocked with 2% donkey serum. The sections were then incubated with the primary antibodies—anti-mouse myeloperoxidase (1:10000; Servicebio) and anti-F4/80 (1:500; Servicebio) overnight at 4°C. To detect NET formation in lung tissue, the sections were incubated with anti-mouse myeloperoxidase and anti-NE (1:500; Bioss Antibodies) overnight at 4°C. Then, the slides were incubated for 2 h with secondary antibodies: Alexa Fluor 488 goat anti-rabbit (1:400; Servicebio) and HRP goat anti-rabbit (1:500; Servicebio). 4′,6-Diamidino-2-phenylindole (DAPI) was used to detect DNA. Finally, slides were visualized using a fluorescence microscope.

### Flow Cytometry Detection

For the quantity and ratio of neutrophils and AMs assay, BALF cell pellets were washed with cold PBS and resuspended in 100 μl of flow buffer. Then, cells were incubated with 1 μM Ly-6G/Ly-6C Monoclonal Antibody (RB6-8C5), PE-Cyanine7 (eBioscience), 1 μM CD80 (B7-1) Monoclonal Antibody (16-10A1), FITC (eBioscience), 1 μM CD170 (Siglec F), and PE (eBioscience) at 4°C for 30 min to allow binding. Upon removal of unbound antibodies by extensive washing of the cells with PBS twice, cells were resuspended in flow buffer and placed in 4°C and blocked from light prior to measurement. All flow-cytometry-based assays were performed on a BD FACS Canto II. Data were analyzed using FlowJo software (Tree Star, Ashland, OR).

### Quantitative Real-Time Reverse Transcriptase Polymerase Chain Reaction (RT-PCR)

mRNA was isolated and cDNA was prepared (Prime-Script RT Master Mix transcription kit, TaKaRa, Tokyo, Japan). RT-PCR was performed to detect NETs and LPS-stimulated MH-S macrophage genes. The RT-PCR contained 4 ng of DNA, isolated as described above, SYBR green master mix (Takara), and primers for IL-1β, IL-6, TNF-α, and β-actin. The enzyme was activated at 95°C for 30 s, followed by 40 cycles at 95°C for 15 s, and 60°C for 60 s. Primer sequences were designed as follows: 5′-TCGCAGCAGCACATCAACAAGAG-3′ (forward) and 5′-TGCTCATGTCCTCATCCTGGAAGG-3′ (reverse) for mouse IL-1β; 5′-TGGGACTGATGCTGGTGACA-3′ (forward) and 5′-ACAGGTCTGTTGGGAGTGGT-3′ (reverse) for mouse IL-6; 5′-ATGTCTCAGCCTCTTCTCATTC-3′ (forward) and 5′-GCTTGTCACTCGAATTTTGAGA-3′ (reverse) for mouse TNF-α; 5′-CTACCTCATGAAGATCCTGACC-3′ (forward) and 5′-CACAGCTTCTCTTTGATGTCAC-3′ (reverse) for mouse β-actin.

### MPO–DNA Complex ELISA

Capture ELISA was used to measure the MPO–DNA complexes as described (14). Briefly, 5 μg/ml anti-myeloperoxidase antibody (Abcam) was diluted and added into an ELISA plate (65 μl per well) overnight 4°C. After blocking with 1% bovine serum albumin, 20-μl BALF samples and the peroxidase-labeled anti-DNA monoclonal antibody (1:25; Roche) were added to each well. The plate was incubated for 2 h in a shaking table at 300 rpm and was washed three times. Finally, 100 μl of peroxidase substrate was added. The absorbance at 405 nm wavelength was measured using BioTek Synergy2 (USA).

### NET Isolation and Quantification

Mouse bone-marrow-derived neutrophils were isolated and purified by density gradient centrifugation as reported (8). Briefly, the bone marrow was flushed out of the tibia and femur using a 25-gauge needle filled with RPMI supplemented with 10% FBS and 2 mM EDTA onto a 50-ml screw top Falcon tube fitted with a 100-μm filter. The red blood cells were lysed by resuspending the cell pellet in hypotonic NaCl solution. The bone marrow cells were resuspended in 1 ml of sterile PBS. Mature neutrophils were purified by centrifugation for 30 min at 2,000 rpm without braking on a Histopaque 1119 and Histopaque 1077. The neutrophils were collected at the interface of the Histopaque 1119 and Histopaque 1077, with typically >95% viable and >95% pure. Purified neutrophils were cultured in free RPMI 1640 medium (Sigma-Aldrich) and stimulated with 500 nM of PMA for 4 h on a 150 × 25 mm flat tissue culture dish. Cell culture supernatants were discarded carefully, leaving the layer of NETs and neutrophils adhered at the bottom. After washing each dish and centrifuging for 5 min at 450 *g* at 4°C, cell-free NET-rich supernatant was obtained. The supernatant was centrifuged for 10 min at 18,000 *g* at 4°C to allow NET pellets to form. The supernatant was discarded and the NET pellets were dissolved in 100 μl of PBS. The purified cell-free NETs were quantified using spectrophotometry. NETs (100–150 ng/μl) were stored at −20°C for subsequent experiments (3).

### Bio-Plex Cytokine Analysis

The BALF and cell culture supernatant levels of IL-1β, IL-6, IL-10, and TNF-α were analyzed using the Bio-Plex Pro Mouse Cytokine 6-plex Assay (Bio-Rad, product No. M6000007NY) according to the manufacturer's instructions in the Bio-Plex 200 system (Bio-Rad, Hercules, CA) as previously reported (15).

### Western Blot Analysis

Primary AMs and stimulated MH-S cells were lysed using RIPA buffer containing a protease inhibitor cocktail (Roche Diagnostics, Indianapolis, IN). The protein concentration was determined using BCA kits (Beyotime Technology). Protein samples (20 μg) were separated by 10% SDS-polyacrylamide gel electrophoresis and subsequently transferred onto PVDF membranes. After blocking with 5% non-fat milk in TBST (TBS with 0.1% Tween-20, pH 7.4) at room temperature for 1 h, the membranes were incubated at 4°C overnight with the following primary antibodies: anti-phospho-ERK1/2 (1:1,000; Cell Signaling Technology), anti-ERK1/2 (1:1,000; Cell Signaling Technology), anti-phospho-NF-κB (1:1,000; Cell Signaling Technology), anti-NF-κB (1:1,000; Cell Signaling Technology), anti-phospho-JNK (1:1,000; Cell Signaling Technology), anti-JNK (1:1,000; Cell Signaling Technology), anti-phospho-p38 (1:1,000; Cell Signaling Technology), and anti-p38 (1:1,000; Cell Signaling Technology). The membranes were washed three times and incubated with HRP-conjugated secondary antibody (CST) at room temperature for 1 h. Anti-GAPDH antibody was used as an internal control. Peroxidase was visualized using an enhanced chemiluminescence system (ECL) (Millipore). Bands were quantitated using ImageJ software (Bio-Rad, USA), and results are expressed as fold change relative to the internal control.

### Statistical Analysis

All data were expressed as the mean ± standard deviation (SD) and statistical analyses were performed using the SPSS version 20.0 (SPSS Inc., Chicago, IL, US). Data with a Gaussian distribution were analyzed by unpaired *t*-test or ANOVA (one-way analysis of variance), while non-parametric data were assessed by Mann–Whitney *U*-test or Wilcoxon rank-sum test. *P* < 0.05 were considered statistically significant.

## Results

### Aerobic Exercise Attenuated Airway Inflammatory Response

**Figure 2:**
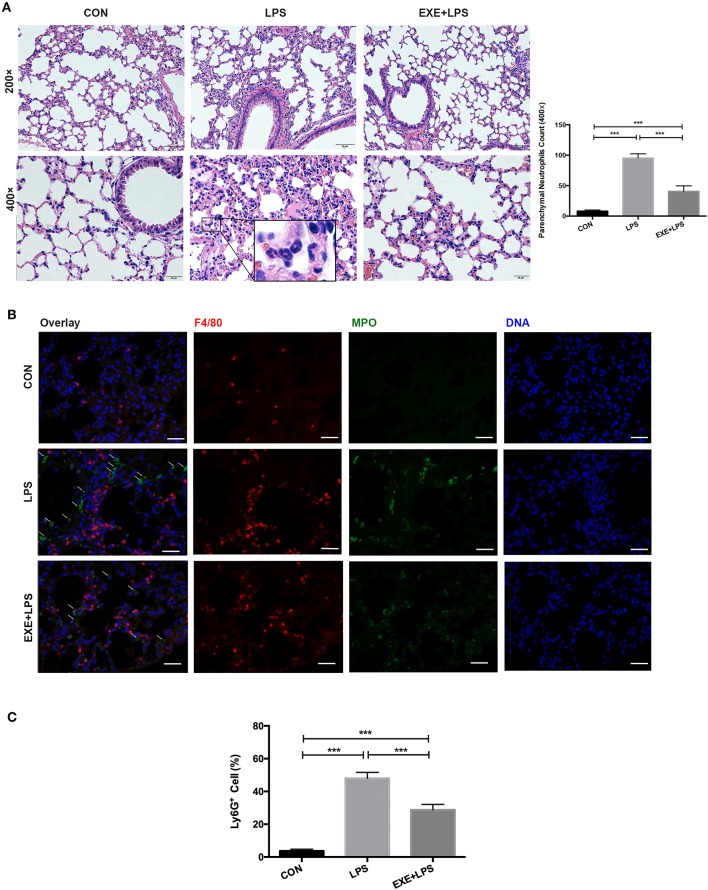
Five weeks of aerobic exercise attenuated LPS-induced airway inflammatory response. **(A)** Images of H&E-stained lung tissue of sedentary mice (left vertical row), 24 h after intraperitoneal LPS injection of sedentary mice (middle vertical row) and 24 h after intraperitoneal LPS injection of exercised mice (right vertical row) (magnification ×200 in the first horizontal row; magnification ×400 in the second horizontal row); the neutrophils are indicated with black arrows in the enlarged black box. The right statistical chart shows parenchymal neutrophils count of three groups at 400× magnification fields. ***p < 0.001. **(B)** Representative images (magnification ×400) show merged DNA (blue), MPO (indicating neutrophils, green), and F4/80 (indicating macrophages, red) via fluorescence microscopy of lung sections from mice in the CON, LPS, and EXE+LPS groups. White arrows show the neutrophil influx. **(C)** The proportion of neutrophils in the BALF of CON, LPS, and EXE+LPS mice was determined with flow cytometry via Ly6G^+^ antibody staining. The statistical chart shows that the percentage of neutrophils in LPS mice was significantly higher than in EXE+LPS mice and CON mice. ***p < 0.001.

Twenty-four hours after the last training bout, LPS and EXE+LPS mice received the same dose of LPS intraperitoneally to establish an extra-pulmonary model of ALI, while CON mice received intraperitoneal PBS. The lung inflammation response was measured through hematoxylin and eosin (H+E) staining of lung parenchyma. The representative photomicrographs revealed a reduced presence of inflammatory cells in the alveolar spaces and in the parenchyma of EXE+LPS mice (Figure 2A) compared to LPS mice. To further clarify the neutrophils influx into the lung parenchyma, we utilized myeloperoxidase (MPO, neutrophil cell marker) and F4/80 (macrophage cell surface marker) to immunofluorescence staining lung tissue and also performed flow cytometry on BALF cells. An immunofluorescence photo of double-labeled lung tissue of EXE+LPS mice revealed a reduced influx of neutrophils (green light) compared to LPS mice (Figure 2B). Also, flow cytometry of the BALF cells showed a reduced proportion of neutrophils in the EXE+LPS mice compared to the LPS mice (Figure 2C).

### Aerobic Exercise and DNase I Treatment Reduce NET Formation and AM Pro-Inflammatory Polarization

**Figure 3:**
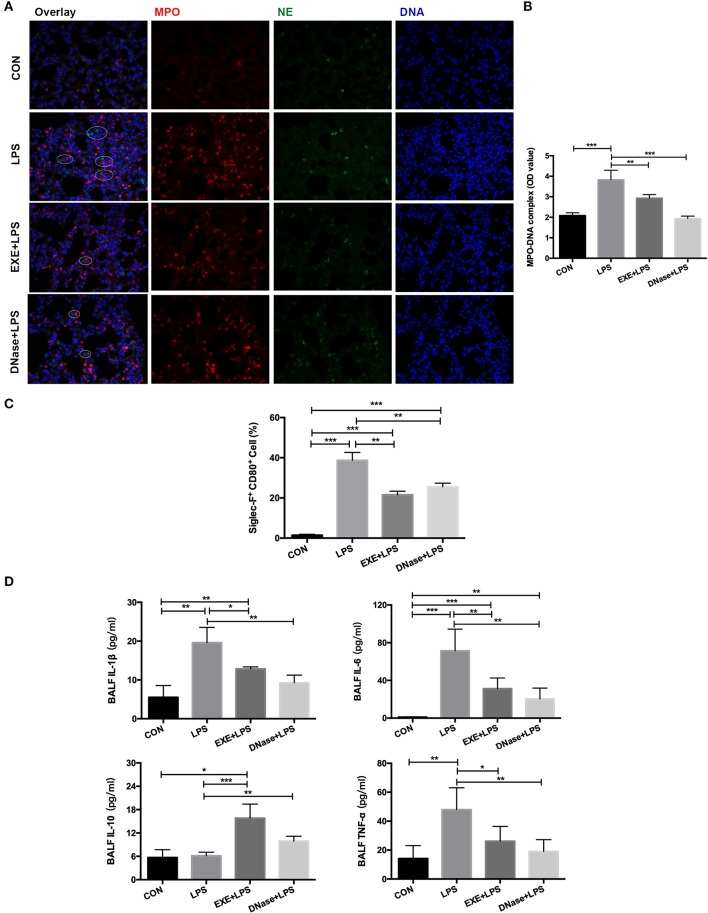
NET formation and alveolar macrophage (AM) polarization with aerobic exercise and DNase I treatment. **(A)** Representative images (magnification ×400) show NET formation of merged DNA (blue), NE (green), and MPO (red) via fluorescence microscopy of lung sections from mice in the CON, LPS, EXE+LPS, and DNase+LPS groups. White circles indicate NETs. **(B)** MPO–DNA complex levels were detected in BALF of the CON, LPS, EXE+LPS, and DNase+LPS mice; the statistical chart shows that the MPO–DNA complex level of the EXE+LPS and DNase+LPS mice was significantly lower than LPS mice. **p < 0.01, ***p < 0.001. **(C)** The pro-inflammatory polarization of AMs in the CON, LPS, EXE+LPS, and DNase+LPS mice were determined via flow cytometry via Siglec-F^+^ and CD80^+^ antibodies staining. The statistical chart shows that the percentage of pro-inflammatory phenotype AMs (Siglec-F^+^ CD80^+^) in the LPS mice was higher than EXE+LPS and DNase+LPS mice. **p < 0.01, ***p < 0.001. **(D)** Pro- and anti-inflammatory cytokines were detected in BALF. The results were expressed as picograms of cytokines per milliliter of BALF. *p < 0.05, **p < 0.01, ***p < 0.001.

NETs were previously reported to participate in the LPS-induced ALI pathological process. Therefore, we explored whether aerobic exercise could reduce the formation of NETs in the injured lung. MPO and NE are the main components of the NET structure. These two immunofluorescent antibodies were stained on the lung tissue. Images from EXE+LPS mice collected by fluorescence microscope displayed less MPO and NE co-localization than with LPS mice, similar to the DNase+LPS mice (Figure 3A). DNase I degrades extracellular DNA structure and effectively destroys the skeleton structure of NETs. A semi-quantitative detection of the MPO–DNA complex on the supernatant of BALF was conducted. The results showed the concentration of MPO–DNA complex in BALF from LPS mice was significantly higher than in EXE+LPS mice (*p* < 0.01) and DNase+LPS mice (*p* < 0.001) (Figure 3B). These results demonstrated that NETs do participate in the LPS-induced ALI process and aerobic exercise can reduce the formation of NETs in the injured lung. Uncontrolled lung inflammation is the most typical feature of ALI and involves polarization of AMs. To examine this phenomenon, the pro-inflammatory macrophage phenotype cell marker (CD80) and the specific cell marker of AMs (Siglec-F) were utilized to detect the proportion of pro-inflammatory AMs (Siglec-F^+^ CD80^+^) in the BALF. The data showed that aerobic exercise and DNase injection significantly reduced pro-inflammatory AMs in the BALF during LPS-induced ALI compared to LPS alone mice (*p* < 0.001) (Figure 3C). At the same time, aerobic exercise and DNase administration reduced BALF levels of inflammatory cytokines including interleukin (IL) IL-1β, IL-6, and tumor necrosis factor alpha (TNF-α) in LPS-induced ALI compared to LPS alone mice (*p* < 0.05 and *p* < 0.01), with an increase in the anti-inflammatory cytokine IL-10 (*p* < 0.001 and *p* < 0.01) (Figure 3D).

### Aerobic Exercise Reduces ALI via Suppressing MAPK and NF-κB Signaling Activation

**Figure 4:**
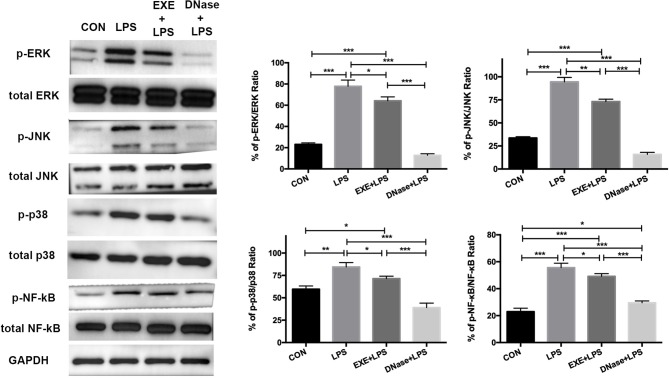
Aerobic exercise and DNase treatment reduces lung inflammation response through MAPK and NF-κB signaling *in vivo*. The total proteins were obtained from primary AMs in the CON, LPS, EXE+LPS, and DNase+LPS mice. Representative Western blots and qualification of phospho-ERK1/2 and total-ERK1/2, phospho-JNK and total-JNK, phospho-p38 and total-p38, and phospho-NF-κB and total NF-κB expression in primary macrophages of all groups. *p < 0.05, **p < 0.01, ***p < 0.001.

These data confirm NET formation and AM pro-inflammatory polarization during LPS-induced ALI. However, the underlying molecular mechanism is obscure. Primary mice alveolar cells were collected from the various groups. After 1.5 h of differential adherence, the pure AMs were lysed to extract total protein. Western blotting results showed significantly increased activation levels of phospho-ERK1/2/ERK1/2 ratio, phospho-JNK/JNK ratio, phospho-p38/p38 ratio, and phosphor-NF-κB/NF-κB ratio in LPS mice compared with CON mice (*p* < 0.0001, *p* < 0.0001, *p* < 0.001, and *p* < 0.0001, respectively) (Figure 4). EXE+LPS mice revealed decreased activation levels both in the phospho-ERK1/2/ERK1/2 ratio, phospho-JNK/JNK ratio, phospho-p38/p38 ratio, and phosphor-NF-κB/NF-κB ratio compared to LPS alone mice (*p* < 0.05, *p* < 0.001, *p* < 0.05, and *p* < 0.05, respectively), while DNase+LPS mice showed the lowest activation levels compared to LPS mice and EXE+LPS mice (Figure 4).

### NETs Promote AM Pro-Inflammatory Polarization via ERK1/2 and NF-κB Signaling *in vitro*

**Figure 5:**
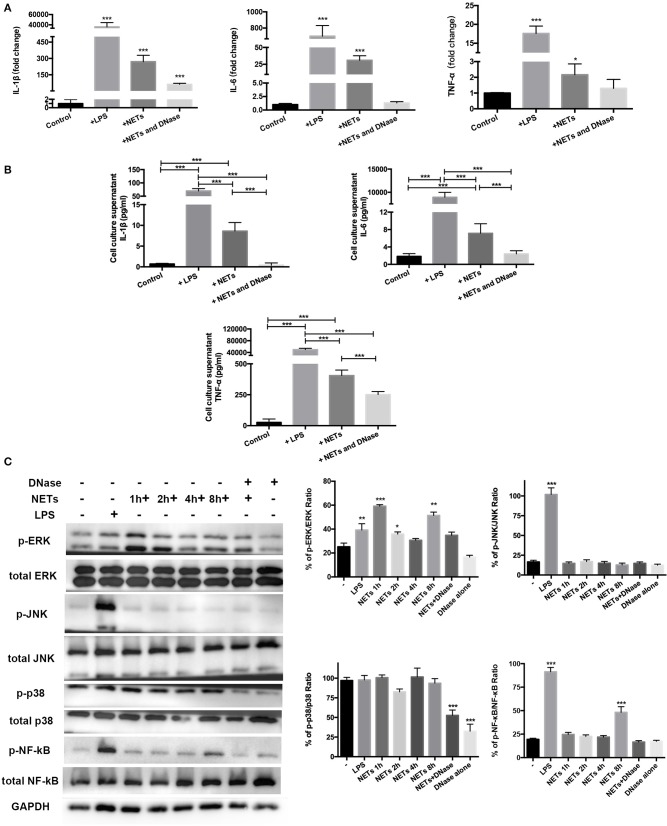
ERK1/2 and NF-κB signaling involved in MH-S cell pro-inflammatory phenotype polarization with NET stimulation *in vitro*. Mice AM cell line MH-S cells were stimulated with 100 ng/ml LPS, 1 ng/μl isolated and purified NETs, and DNase pre-treated NETs for 8 h. **(A)** IL-1β, IL-6, and TNF-α expression in these stimulated MH-S cells was measured using PT-PCR, indicating the degree of pro-inflammatory phenotype polarization of the stimulated cells compared with the unstimulated cells. **(B)** IL-1β, IL-6, and TNF-α expression in the cell culture supernatant was determined using Bio-Plex cytokine analysis. **(C)** Representative Western blots and qualification of phospho-ERK1/2 and total-ERK1/2, phospho-JNK and total-JNK, phospho-p38 and total-p38, phospho-NF-κB, and total NF-κB expression in primary macrophages of control cells, LPS-stimulated cells, NETs+DNase-stimulated cells, DNase alone-stimulated cells for 8 h, and NET-stimulated cells for 1, 2, 4, and 8 h. All stimulated cells were statistically compared to unstimulated cells. *p < 0.05, **p < 0.01, ***p < 0.001.

The data supported involvement of MAPK and NF-κB signaling pathways in mediating exercise-induced attenuation of the ALI inflammation response. Isolated and purified NETs were used to directly stimulate AMs *in vitro* to determine if a decrease in NETs influenced AM polarization. MH-S, a cell line of mice AMs, was stimulated with 100 ng/ml LPS, 1 ng/μl NETs, and DNase I pre-treated NETs for 8 h. The cell culture supernatant was collected for cytokine detection and cells were lysed for RT-PCR and Western blot analysis. RT-PCR results showed that LPS, NETs, and NETs+DNase stimulation all significantly increased IL-1β expression (*p* < 0.001) of MH-S cells compared to control while NETs+DNase stimulation showed the smallest increase (Figure 5A). IL-6 and TNF-α expression of MH-S cells were higher when stimulated with LPS and NETs (*p* < 0.001 and *p* < 0.001/0.05) compared to control. NETs+DNase stimulation showed no difference compared to control (Figure 5A). Cell culture supernatant levels of IL-1β, IL-6, and TNF-α revealed similar trends between the four groups. Concentrations of the four cytokines were higher under LPS compared to NET stimulation, and significantly higher compared with control (*p* < 0.001). NETs+DNase stimulation showed no difference with control (Figure 5B). These results support a catalytic effect of NETs on AMs pro-inflammatory phenotype polarization. To explore underlying molecular and signaling mechanisms, Western blotting of the MH-S cells was conducted. The data revealed significantly increased levels of phospho-ERK/ERK ratio after 1, 2, and 8 h NET stimulation (*p* < 0.001, *p* < 0.05, and *p* < 0.01). The phosphor-JNK/JNK and phosphor-p38/p38 ratios were unaffected after NET stimulation. The phosphor-NF-κB/NF-κB ratio increased after 8 h of NET stimulation (*p* < 0.001) (Figure 5C). Phosphor-ERK1/2, JNK, and NF-κB, but not p38, protein activations were involved in LPS-induced MH-S cell pro-inflammatory responses (Figure 5C).

## Discussion

Our study demonstrated that 5 weeks of aerobic exercise training reduced the inflammatory response to LPS-induced ALI in mice by decreasing the excessive release of NETs and AM pro-inflammatory polarization. The beneficial influence of aerobic exercise on LPS-induced lung inflammation has been reported previously (16). These data from the current study extend these findings by improving understanding of underlying mechanisms.

The beneficial effect of aerobic exercise training on LPS-induced ALI and NET production was similar to the therapeutic effect of DNase injection. In order to cope with the invasion of pathogenic microorganisms, the body needs to find a balance between infection control and the immune response to avoid collateral damage to host tissues (17). A key feature of the LPS-induced ALI syndrome is an early, robust neutrophilic response (18). Infiltration of neutrophils and the release of NETs play a role in controlling lung infections, but at the same time, excessive formation of NETs inevitably aggravates the extent of lung injury. Our data demonstrate that 5 weeks of aerobic exercise training can help the body achieve the appropriate balance between pulmonary infection control, tissue homeostasis, and the immune response. Physical exercise improved histological and oxidative stress parameters in mice exposed to cigarette smoke (19). Exercise enhanced the activity of antioxidant enzymes (20) and up-regulated the expression of genes related to energy pathways and lipid metabolism pathways (21, 22). Additionally, exercise training reduced epithelial proliferation and mucin expression, and inhibited endothelium-dependent vasorelaxation dysfunction induced by various air pollutants in the lungs (23, 24). These findings are in good agreement with the anti-inflammatory role of exercise training in pulmonary diseases and provide evidence of exercise-mediated prevention of LPS-induced airway inflammation.

NETs have been explored extensively in the area of lung disease as contributing to the defense against bacterial respiratory infections (25). Bacterial entrapment by NETs impedes the ability of pneumococci to spread from the upper to the lower respiratory tract and to disseminate into the bloodstream (16). Mice with increased NET formation caused by a deficiency in the anti-inflammatory adenosine A2B receptor have been shown to have an enhanced capacity for bacterial clearance from the lungs and increased survival when challenged with Klebsiella pneumonia (26). Although NETs have potentially beneficial anti-pathogenic functions in respiratory host defenses, protection can easily turn to injury when extensive, unbalanced NET formation occurs. NETs are embedded with potent granule proteins that are capable of killing pathogens but may also, under the right conditions, induce cell death of lung epithelial and endothelial cells (27) and contribute to certain lung diseases such as asthma (28), cystic fibrosis (29), and COPD (30). Little is known regarding the interaction between NETs and specific cells, including AMs. Recently, Song et al. (11) reported that NET levels in ARDS patients were positively correlated with M1-like macrophage polarization. Hu et al. (31) also demonstrated that NET formation may be involved in the activation of pro-inflammatory macrophages in Adult-onset Still's disease patients. These data contributed to the design of this study, with the aim of further exploring the role of NETs on macrophages. Our *in vivo* results showed a potential relationship between NETs and pro-inflammatory phenotype polarization of lung AMs. We then showed *in vitro* that extracted NETs stimulated mouse-derived AMs (MH-S) to develop in the direction of a pro-inflammatory phenotype. To a certain extent, this finding further clarifies the possible mechanism of the adverse effect of excessive NET formation in aggravating pulmonary inflammation during ALI.

LPS-induced ALI has been related to the MAPK pathways, including the extracellular signal regulated kinases ERK1/2 (32), p38, and JNK (33). These can be initiated by LPS-TLR4 receptor activation (34), leading to gene transcription and pro-inflammatory cytokine production. Others have shown that CXCR4 knockdown prevents inflammatory cytokine expression in macrophages by suppressing activation of MAPK and NF-κB signaling pathways (35). A chief aim of the current study was to explore the mechanism by which aerobic exercise training improves ALI using murine primary AMs. We found that the expression level of phosphorylated ERK1/2, JNK, p38 proteins, and phosphorylated NF-κB proteins of EXE+LPS mice was significantly suppressed when compared to the LPS mice. The expression level of these proteins was lowest in the sedentary group receiving DNase intervention (DNase+LPS group). These data provide evidence that aerobic exercise attenuates LPS-induced ALI by inhibiting NF-κB and MAPK signaling pathways, and that NETs may play a crucial role in this process. Given the pro-inflammatory effects of NETs on AMs, we sought to confirm the NET-associated molecular mechanisms by Western blot analysis. We detected a significant increase in the phosphorylated forms of both ERK1/2 and NF-κB pro-inflammatory proteins in MH-S cells when stimulated with NETs, with no effect on p38 and JNK proteins. Interestingly, the phosphorylation of ERK1/2 protein increased in the early phase of NET stimulation (at 1 and 2 h) during the 8-h period of stimulation of MH-S cells. The phosphorylated NF-κB protein ratio increased in the late stages of stimulation (at 8 h). These data suggest that NETs exert an activation of signaling pathways in a defined sequence during MH-S cell stimulation. The *in vivo* and *in vitro* data support that three key MAPK signaling pathway proteins (ERK1/2, JNK and p38) and NF-κB protein were inhibited with aerobic exercise training, and helped attenuate LPS-induced ALI. However, the *in vitro* data suggest that MH-S cells turned to M1 phenotype under NET stimulation, with an activation of ERK1/2 protein and NF-κB proteins. Thus, aerobic exercise training may reduce the production of NETs by inhibiting the ERK1/2 and NF-κB pathways, leading to a reduction in M1-type polarization of AMs and the inflammation response of ALI.

There are several limitations in our study procedure and data. We demonstrated to some extent that NETs play a key role in the attenuation of LPS-induced ALI with aerobic exercise training. However, we did not establish a direct causal relationship between these by blocking neutrophils *in vivo* or knocking out key genes that mediate NET release such as peptidylarginine deiminase 4 (PAD4) (36). Secondly, the molecular mechanism by which NETs stimulates the polarization of AMs to pro-inflammatory phenotype needs additional analysis. We verified the involvement of key proteins from the MAPK and NF-κB signaling pathways, but many others could be involved.

In conclusion, our study revealed that 5 weeks of aerobic treadmill running attenuated LPS-induced ALI in mice through inhibition of NET formation and AM pro-inflammatory phenotype polarization via suppression of ERK1/2 and NF-κB signaling pathways.

## Data Availability Statement

All datasets generated for this study are included in the article/supplementary material.

## Ethics Statement

The animal study was reviewed and approved by the Ethics Committee of Shanghai University of Sport.

## Author Contributions

PC, YS, and HS contributed to the conception and design of the study. LY, YS, and FL performed the animal training program. YS and TL performed the *in vivo* experiments and cell culture studies. YS, TL, YC, and HS performed the results analysis. YS and HS prepared the figures and drafted the article. DN and PC edited and revised the article.

### Conflict of Interest

The authors declare that the research was conducted in the absence of any commercial or financial relationships that could be construed as a potential conflict of interest.
